# Individual- and Household-Level Interventions to Reduce Air Pollution Exposures and Health Risks: a Review of the Recent Literature

**DOI:** 10.1007/s40572-020-00296-z

**Published:** 2020-11-26

**Authors:** Ryan W. Allen, Prabjit Barn

**Affiliations:** 1 Faculty of Health Sciences, Simon Fraser University, Burnaby, BC Canada; 2 Legacy for Airway Health, Vancouver Coastal Health, Vancouver, BC Canada

**Keywords:** PM2.5, Smoke, Traffic, Intervention, Randomized, Crossover, HEPA, Mask, N95, Respirator

## Abstract

**Purpose of Review:**

We reviewed recent peer-reviewed literature on three categories of individual- and household-level interventions against air pollution: air purifiers, facemasks, and behavior change.

**Recent Findings:**

High-efficiency particulate air/arresting (HEPA) filter air purifier use over days to weeks can substantially reduce fine particulate matter (PM_2.5_) concentrations indoors and improve subclinical cardiopulmonary health. Modeling studies suggest that the population-level benefits of HEPA filter air purification would often exceed costs. Well-fitting N95 and equivalent respirators can reduce PM_2.5_ exposure, with several randomized crossover studies also reporting improvements in subclinical cardiovascular health. The health benefits of other types of face coverings have not been tested and their effectiveness in reducing exposure is highly variable, depends largely on fit, and is unrelated to cost. Behavior modifications may reduce exposure, but there has been little research on health impacts.

**Summary:**

There is now substantial evidence that HEPA filter air purifiers reduce indoor PM_2.5_ concentrations and improve subclinical health indicators. As a result, their use is being recommended by a growing number of government and public health organizations. Several studies have also reported subclinical cardiovascular health benefits from well-fitting respirators, while evidence of health benefits from other types of facemasks and behavior changes remains very limited. In situations when emissions cannot be controlled at the source, such as during forest fires, individual- or household-level interventions may be the primary option. In most cases, however, such interventions should be supplemental to emission reduction efforts that benefit entire communities.

## Introduction

Air quality improvements in wealthy countries have improved public health [1–4]. Global progress toward clean air, however, has been uneven. More than 90% of the world’s population breathes fine particulate matter (PM_2.5_) above the World Health Organization’s (WHO) guideline concentration and air pollution remains a leading cause of disease and premature death [1]. Even low concentrations pose a threat because there appears to be no safe exposure threshold [5–7].

It will be decades before the global health impacts of air pollution can be reduced to acceptable levels [8]. For example, between 2013 and 2017, China’s ambitious Air Pollution Prevention and Control Action Plan reduced PM_2.5_ concentrations by 33% across 74 cities [9–11]. But following that extraordinary improvement, the average PM_2.5_ concentration in those cities was 47 μg/m^3^—or nearly five times the WHO guideline concentration [10]. We have worked for several years in Ulaanbaatar, Mongolia, a city with notoriously polluted air [12–16]. Piecemeal efforts by the government and international organizations to reduce pollution have had little impact, and citizens have protested and demanded that more be done [17]. Clearing the air will require radical changes to the city’s energy supply and infrastructure, so even under optimistic scenarios Ulaanbaatar’s air will threaten public health for many years to come [18].

Given the slow pace of progress in many settings, individuals struggle to protect themselves and their communities [19–21]. A private campaign in Ulaanbaatar raised approximately $40,000 (US) in financial and in-kind support to distribute air purifiers to children’s hospital wards [22], and we frequently hear stories of families sending children to schools abroad to escape the pollution. Similarly, some wealthy “pollution refugees” are leaving other highly polluted cities like New Delhi for places with cleaner air [23].

Some individual- and household-level (IHL) interventions are now big business, although the marketing claims often exaggerate the scientific evidence [24–26]. One can purchase “DIY” air purifiers kits [27], air filtering baby pillows are being developed [28], and facemasks appear at fashion shows [29]. During a particularly polluted period in New Delhi, officials announced plans to distribute five million facemasks to schoolchildren [30]. One study estimated that a day of “severe” pollution in eight Chinese megacities leads to $200,000 (US) in facemask sales [31]. Another study estimated that a 10% reduction in the number of “heavily polluted” days across China over a 16-month period would reduce facemask sales by $187 million (US) [32].

IHL interventions represent a “Band Aid” solution and are not a viable long-term alternative to emissions reductions. But some of these interventions may have value—particularly in high pollution settings, for vulnerable individuals, and/or when emissions cannot be managed at the source.

Interest in these interventions among researchers continues to grow. Several reviews and commentaries have been published in recent years [33–42], with most discussing one class of interventions [36•], a narrow range of health outcomes [37, 41], a specific pollution source [42], and/or specifically targeting clinicians [38–41]. We sought to broadly summarize the most important recent research findings for three categories of interventions: air purifiers, facemasks, and behavior modifications. Our goal was not to conduct a systematic review, but rather to identify contemporary research themes and areas of overlap, offer suggestions for future studies, and share some thoughts based on our own research experiences in this area.

We searched the PubMed and Web of Science databases by combining search terms related to air pollution (e.g., particulate matter, PM_2,5_, smoke) with terms related to our three categories of interventions (e.g., HEPA, mask, windows). We reviewed the abstracts identified by these searches, selected relevant articles, and then performed reference and citation searches on them. We were primarily interested in intervention studies targeting PM_2.5_, the pollutant most consistently linked with health outcomes, and studies published in 2017 or later (although some particularly relevant older studies are discussed). Interventions targeting air pollution from cooking and/or heating stoves are outside our scope and are discussed elsewhere [43, 44].

## Air Purifiers

People spend most of their time indoors [45]. Because outdoor air pollution infiltrates into buildings, “indoor air” contains both indoor- and outdoor-generated pollution [46]. Indoor exposures account for 61% and 81% of the deaths attributed to outdoor-generated PM_2.5_ in the USA and China, respectively [47, 48]. Thus, reducing PM_2.5_ indoors may also mitigate the impacts of outdoor-generated particles.

Indoors, particles can be removed by placing air purifiers in a building’s heating, ventilation, and air conditioning (HVAC) system or by using portable, stand-alone air purifiers. Most studies have focused on mechanical filtration with high-efficiency particulate air/arresting (HEPA) filters, which remove at least 99.97% of 0.3 μm particles. Fewer studies have evaluated other technologies such as electrostatic precipitators (EPs) or negative ion generators (IGs), both of which produce ions that attach to particles and promote deposition. Two factors govern air purifier effectiveness: the efficiency of the device at removing pollution and the volume of air brought through the device. Two rating systems are commonly used to evaluate air purifiers. HVAC filters are given a minimum efficiency reporting value (MERV) between 1 and 16, while portable air purifiers are often described by the clean air delivery rate (CADR). More details on air purification technologies were provided in a recent review [36•].

By 2017, there was substantial evidence from randomized studies in North America and Europe that HEPA filtration—either in HVAC systems or with portable units—can reduce PM_2.5_ concentrations by 50% or more over periods of a few days to 2 weeks [36•]. Several studies also reported improvements in subclinical cardiovascular health indicators including systolic blood pressure [49], endothelial function [50, 51], and systemic inflammation [51, 52]. Our review identified four main themes in recent research: (1) evaluations of exposure and health impacts from air filtration in highly polluted settings and over longer durations, (2) studies of personal exposure impacts from filtration, (3) cost-benefit analyses of filtration, and (4) investigations involving EPs and IGs (Table 1).

Three recent studies in China demonstrated large concentration reductions from air filtration in highly polluted settings. In Beijing, portable HEPA filter air purifier use for 2 weeks reduced PM_2.5_ concentrations from 60 to 24 μg/m^3^ in the homes of non-smoking seniors [53, 54]. The air purifiers also reduced systemic inflammation, but did not affect lung function, blood pressure, or heart rate variability (HRV) [53]. In a suburb of Shanghai, 13 h of portable HEPA filtration reduced mean indoor PM_2.5_ concentrations from 33 to 10 μg/m^3^ in the homes of non-smoking young adults [55]. Filtration was associated with improved airway mechanics and reduced thrombosis risk, with no improvements in spirometry or airway inflammation—and a small, marginally significant increase in diastolic blood pressure. In a study of young adults in Shanghai, use of portable air filters over 9 days reduced average PM_2.5_ concentrations in dormitories from 47 to 9 μg/m^3^, while also reducing stress hormones and improving indicators of blood pressure, insulin resistance, oxidative stress, and inflammation [56].

Two recent studies evaluated air purifier efficacy over relatively long durations. Chuang and colleagues [57•] enrolled 200 homemakers in Taipei into a study of MERV 11 filtration in window air conditioning units. Participants were randomly assigned to a filtration intervention or sham control group for 1 year, and then to the opposite group for the second year. Filters reduced indoor PM_2.5_ concentrations from 22 to 13 μg/m^3^ and were associated with reductions in blood pressure, systemic inflammation, and oxidative stress. Our Ulaanbaatar Gestation and Air Pollution Research (UGAAR) study is a randomized controlled trial designed to evaluate the impacts of portable HEPA filter air purifier use during pregnancy on fetal growth and childhood development [58•, 59]. Air purifiers reduced indoor PM_2.5_ concentrations during pregnancy by 29% (from 25 to 17 μg/m^3^), with greater PM_2.5_ reductions in colder months. In addition, effectiveness was greater when air purifiers were first deployed (40% reduction) than after approximately 5 months of use (15% reduction). Overall, participants reported using air purifiers for 64% of the study period. The intervention led to an 85 g (95% CI: 3, 167 g) increase in mean term birth weight.

Three recent studies suggested that air filters have less impact on personal exposures than on indoor concentrations. In Detroit, Morishita and colleagues [60] measured PM_2.5_ exposure and blood pressure changes among 40 non-smoking participants in a low-income senior residential facility. Air purifiers, which were placed in participants’ bedrooms and living rooms, reduced indoor PM_2.5_ concentrations by 52% with low efficiency filtration and by 59% with HEPA filtration. Personal PM_2.5_ exposure reductions were smaller: 30% with low efficiency filtration and 52% with HEPA filtration. The authors did not report the amount of time participants spent inside their homes. Air filtration was associated with significant reductions in systolic blood pressure and marginally significant reductions in diastolic blood pressure. In Beijing, Zhan et al. [61] found that 48 h of portable HEPA filtration in six residences reduced average bedroom PM_2.5_ concentrations from 49 to 9 μg/m^3^, but average personal exposure was *higher* during filtration. The authors attributed the lack of exposure reductions to time spent in other microenvironments. A crossover study of 43 children near Shanghai indicated that 2 weeks of bedroom HEPA filtration reduced average indoor PM_2.5_ concentrations by 68% but reduced average personal exposure by only 27%. Although the children’s time-activity patterns were not reported, the authors attributed the more modest reductions in personal exposure to time spent in other locations [62].

Three recent studies suggest that the economic benefits of filtration would often exceed purchase and operation costs, particularly if the filtration is targeted to susceptible subpopulations [63, 64•, 65]. Fisk and Chan [63] modeled the impacts of portable and HVAC air filter use for 10 days during the 2003 southern California wildfires. When used in all homes, the economic benefits of reduced mortality outweighed costs for HVAC filtration, but not for portable air purifiers. However, both interventions were more cost effective when targeted elderly residents, a group at elevated risk of mortality during fire smoke events. In a second study, these authors quantified the costs and economic benefits of reduced particle-related mortality from long-term user of portable and HVAC filtration in US homes and workplaces [64•]. Benefits exceeded costs in all scenarios, with cost-benefit ratios ranging from 3.9 to 133. Investigators in Detroit modeled the impact of enhanced HVAC filtration in homes and schools on exposure to outdoor-generated PM and asthma-related outcomes [65]. They found that installing enhanced filters in schools would be cost effective, while enhanced filters in homes of asthmatic children would be more expensive and less cost effective. These studies probably underestimated total benefits because they focused on a narrow range of health outcomes and only considered the impacts of outdoor-generated pollution.

Finally, three recent studies reported potentially determinantal effects from other air purifier technologies. In China’s Hunan province, removal of HEPA filters from HVAC systems in offices and dormitories led to large increases in PM_2.5_ concentrations but did not alter biomarkers of cardiopulmonary risk among 89 young adults [66]. But removal of EPs from HVAC systems led to small *decreases* in ozone concentrations and several cardiovascular risk biomarkers, indicating potential adverse effects from ozone produced by EPs. In Beijing, Liu and colleagues [67•] conducted a randomized crossover study of IGs in the dormitories of 56 healthy university students. One week of IG use reduced indoor PM_2.5_ concentrations but increased negative ion concentrations. Negative ions were associated with an increase in malondialdehyde, a biomarker of systemic oxidative stress, which offset the benefits of PM_2.5_ reductions resulting in no net effect of IG use on malondialdehyde. There were also no effects of IG use on measures of lung function, vascular tone, arterial stiffness, or inflammation. In a similar study of 44 children in Beijing classrooms, Dong et al. [68] reported that 5 days of IG use decreased PM_2.5_ and BC, improved lung function and reduced airway inflammation, but did not alter blood pressure. However, the study also found that negative ions were associated with detrimental effects on HRV.

## Facemasks

Unlike air purifiers, facemasks have the potential to reduce exposure in multiple indoor and outdoor locations. We use the term “facemask” to include a range of materials worn on the face including improvised masks (t-shirts, handkerchiefs, bandanas, etc.) [69], procedure or surgical masks, and air purifying respirators designed for occupational settings [70].

Occupational health and safety organizations typically evaluate and certify respirators used in workplaces [71]. For example, in the USA, the National Institute for Occupational Safety and Health categorizes particle-filtering respirators by three categories of oil resistance and three levels of efficiency (95, 99, and 99.97%) at removing 0.3 μm particles [71]. The most common of these is the N95 (or equivalent, e.g., FFP2 in Europe and KN95 in China) [72]. Facemask effectiveness is governed by both the material’s filtration efficiency and the mask’s facial fit, and in workplaces respirators are typically fit tested on workers. In contrast, respirators marketed for use in community settings do not typically undergo testing or certification. The costs of facemasks vary widely. Typical costs for procedure masks and N95 respirators are < $0.50 US and $2 US, respectively, while reusable respirators marketed for protection against community air pollution can cost up to $50 US.

To our knowledge, only two studies published prior to 2017 evaluated the health benefits of facemask use in community settings [73, 74]. Both were conducted in Beijing and provided evidence that short-term use of N95 respirators can reduce the effects of air pollution on blood pressure and HRV. Recent studies have added to this limited literature on health impacts, while others evaluated the exposure benefits of a broader range of facemask materials and designs.

Since 2017, three additional studies of N95 masks and health outcomes have been published. In Shanghai, Shi and colleagues [75] conducted a randomized crossover trial in which 24 healthy young adults wore N95 respirators during one of two 48-h periods. Participants were instructed on how to wear respirators to achieve a good fit. Respirator use was associated with reduced mean systolic blood pressure and improved HRV. Yang and colleagues [76] performed a randomized crossover study in Beijing’s subway system to evaluate short-term cardiovascular benefits from both respirators and noise-canceling headphones. Participants underwent extensive respirator fit testing. Both interventions were associated with improved HRV and decreased heart rate, but there were no associations with blood pressure. Most recently, Guan and collaborators [77•] studied the influence of N95 masks on airway inflammation and endothelial function among 15 healthy young volunteers in Beijing. The notable difference from previous work was the double-blind design with sham facemasks (facemasks with filters removed). The authors did not provide information on fit testing. After participants walked a defined route along a busy road for 2 h while wearing a facemask, the investigators measured health outcomes four times over a 24-h period. The procedure was repeated 1 month later using the opposite mask configuration. Compared with sham facemasks, the real facemasks attenuated pollution-induced effects on airway inflammation. There were no effects on systemic oxidative stress, and one arterial stiffness indicator was significantly *elevated* after participants wore the real facemasks. This may have been due to facemasks increasing respiration resistance or inducing deeper breathing leading to changes in airway deposition.

Several other recent studies of facemasks evaluated the exposure reduction potential of various facemask materials and designs. Two studies used mannequins and two enrolled human volunteers, but all emphasized the importance of facial fit on performance. Using a mannequin, Shakya and colleagues [78] found that cloth masks filtered 15 to 57% of diesel particles. A surgical mask had 79% efficiency, similar to the N95 masks tested. The authors noted that the best performing cloth mask conformed to the mannequin’s contours while the less effective cloth masks did not provide a good fit. Pacitto et al. [79•] studied nine facemasks available in Spain at prices ranging from 1 to 44 Euros (approximately $1.10 to $50 US). Mask effectiveness in removing PM_2.5_ ranged from 14 to 96%, with lower effectiveness for black carbon and particle number count. The authors noted that mask costs were related more closely to esthetics than effectiveness.

Cherrie and colleagues [26] evaluated particle penetration through nine facemasks that are commercially available in China, then selected four masks for additional testing on human volunteers. A key finding was that despite being made of highly efficient particle-filtering materials, two of the four masks performed poorly during human volunteer testing because of inadequate fit. A similar study evaluated 17 facemasks—ranging in sophistication from improvised masks to an N99 mask—used for protection against volcanic ash, which is composed of larger and more easily filtered particles than urban air [69]. Certified masks (N95 and N99) performed best (filtration efficiencies > 98%), followed by surgical masks (89–91%) and improvised mask materials (< 44%). Four of the masks—including an N95 respirator, a surgical mask designed to filter PM_2.5_, a standard surgical mask, and a basic “flat fold” dust mask—were then tested on human volunteers for quantification of total inward leakage (TIL) [80]. The TIL of the N95 mask was 9%, compared with 22 to 35% for the other masks. Study participants rated the N95 as the most protective due to sturdiness and fit, but also as uncomfortable and difficult to breathe through.

## Behavior Modifications

### Staying Indoors and Closing Windows

Air pollution dose is influenced by concentration and breathing rate, so changing locations and activities may be beneficial [33]. During pollution episodes, government and public health agencies often recommend reducing outdoor activities, particularly for susceptible groups [81, 82].

The level of protection provided by residences and other buildings varies depending on window opening, air conditioning use, use of air purifiers, building age and condition, and other factors. In seven US communities, we found mean PM_2.5_ infiltration efficiencies (*F*_inf_, the fraction of outdoor PM_2.5_ concentration that penetrates indoors and remains suspended) ranging from 0.49 to 0.74 in the heating season and 0.43–0.90 in the non-heating season [46]. Several recent studies in Chinese cities estimated mean *F*_inf_ in the heating and non-heating seasons of 0.54–0.79 and 0.70–0.91, respectively [83–85].

To our knowledge, only two studies—both conducted in Taipei—directly evaluated the health benefits of keeping windows closed and both were published prior to 2017. In a 2009 study, 40 healthy university students underwent hourly blood pressure and heart rate measurements during four 48-h monitoring sessions [86]. The researchers asked participants to stay home and keep gas stoves turned off, and to keep windows open during the first two measurements and closed during the last two measurements. Indoor PM_2.5_ concentrations, blood pressures, and heart rates were higher when windows were open than when windows were closed. The same researchers later enrolled 300 healthy adults and compared three exposure conditions: windows open, windows closed without air conditioning, and windows closed with air conditioning [87]. Closing windows had little effect on PM_2.5_ concentrations, but levels were 44% lower when air conditioning was used (26 vs 14 μg/m^3^). HRV improved both when windows were closed and when air conditioning was turned on, while oxidative stress and systemic inflammation markers decreased only when air conditioners were turned on. Unfortunately, the investigators did not randomize treatment order or report outdoor PM concentrations.

In a 2019 study, Reisen and colleagues [88] reported measurements of outdoor and indoor PM_2.5_ concentrations at 21 residences in Australian community impacted by smoke from prescribed burns and bushfires. Seven of the homes were monitored during smoke plume events. There were no personal exposure measurements. Residents completed a diary on indoor pollution source activities and window opening. The authors found that during smoke plume events hourly peak PM_2.5_ concentrations were reduced by 12 to 76% when windows and doors were closed. But the authors also reported that leaving windows closed after a smoke event had ended trapped PM_2.5_ indoors and increased indoor concentrations.

The potential protection provided by buildings must be balanced with other factors including indoor pollution sources [89], heat [90], and impacts on physical activity [91•]. A 2018 study in Beijing estimated that increasing building air-tightness, without adding mechanical ventilation and/or filtration, would reduce the infiltration of outdoor pollution but amplify the impact of indoor emissions, leading to higher indoor PM_2.5_ concentrations [89].

### Travel Modes and Routes

Travel can disproportionately impact exposure to traffic-related air pollution (TRAP) [92, 93]. TRAP concentrations are highly variable and several cities have online tools to help commuters select routes with less air pollution. Travel mode can also be important because it influences travel route and duration, breathing rate, and the degree of protection from ambient pollution.

Three review papers published in 2017 or 2018 concluded that, compared to active commuters, drivers generally encountered higher concentrations of TRAP [91, 94, 95]. However, active commuters often had higher inhaled doses due to increased minute ventilation and longer trip durations [91•, 95]. Incorporating the benefits of physical activity, Cepeda and colleagues [91•] found that median life expectancy losses were up to 1 year lower among active commuters, indicating that the benefits outweighed the negative effects of pollution.

There were several randomized crossover studies of route and cardiopulmonary health published prior to 2017. Results from studies of cyclists were mixed [96–99], while a study of individuals with asthma in London found that walking for 2 h on Oxford Street induced greater reductions in lung function than walking in Hyde Park, where pollution levels were lower [100].

Our review identified three recent crossover studies of travel routes and health in addition to several recent studies of exposure. Participants in London aged 60 years and older walked for 2 h either along Oxford Street or in Hyde Park, then in the opposite location 3 to 8 weeks later [101]. Personal exposures to TRAP and noise were significantly higher on Oxford Street. Walking in Hyde Park led to improvements in several cardiopulmonary markers, while walking on Oxford Street was less beneficial. In a study of 32 healthy adult cyclists in Sacramento, Park and colleagues [102] asked participants to complete one ride each on a low- and high-traffic route. UFP concentrations were nearly three times higher on the high-traffic route. Lung function measures improved while cycling on the low-traffic route and either decreased or were unchanged during the high-traffic ride. Unfortunately, the specific routes were not uniform across participants and the authors did not indicate whether route order was randomly assigned. In Vancouver, Cole et al. [103] conducted a randomized crossover study in which 38 adults cycled for 1 h on both a downtown and residential route. Median exposures to PM_2.5_ and UFP were 30% and 53% higher, respectively, on the downtown route. Reactive hyperemia index (RHI, a measure of endothelial function) decreased after cycling downtown and increased after cycling in the residential area. But changes in RHI were not associated with air pollutants and the authors speculated that the differences between routes may have been due to other exposures such as physical exertion, noise, or stress. Other measures of lung function, systemic inflammation, and oxidative stress were not associated with route.

Several other recent studies reported on the effect of route on TRAP exposure only, with most focused on cyclists. These studies consistently found routes with less vehicle traffic were associated with lower BC concentrations, with less consistent evidence of reductions in UFP or PM_2.5_ [104–110]. A study in Fort Collins, Colorado, a city with relatively low levels of air pollution, also found that by selecting an alternative route drivers significantly reduced cumulative exposures to BC and carbon monoxide by 15%, but PM_2.5_ exposures were unchanged [110].

### Vehicle Ventilation and Filtration

By 2017, there was substantial evidence that in-vehicle concentrations of UFP and other traffic-related particles are influenced by opening windows, recirculating cabin air, and/or the use of enhanced filtration [111–115]. These findings have been reinforced by recent studies, some of which documented considerable variation in particle concentrations under different ventilation conditions [115–117]. For example, Kumar and colleagues [117] drove pre-planned routes in 10 cities and measured in-vehicle PM_2.5_ concentrations with windows open and fan off (windows open), windows closed with fan on (fan on), and windows closed with recirculation mode on (recirculation). Relative to recirculation mode, PM_2.5_ concentrations were up to 385% higher with fan on and up to 1020% higher with windows open.

In contrast, the evidence that vehicle ventilation or filtration influence health is much more limited. Prior to 2017, we are aware of one study that directly addressed this issue. In that non-randomized study, the use of a vehicle’s air conditioning system improved air quality and modified the effects of PM_2.5_ on HRV [118]. More recently, in a non-randomized study, Yu and colleagues [119, 120] found that using high-efficiency cabin air filters in taxis with windows closed reduced mean PM_2.5_ and ultrafine concentrations by 37% and 47%, respectively, but did not alter oxidative stress indicators.

That study and several others published recently also emphasized the trade-offs between closing windows and recirculating cabin air to reduce particle concentrations and avoiding high levels of CO_2_, which can cause drowsiness and cognitive impairment [115, 120–125]. Hudda and Fruin [125] conducted a modeling study to identify vehicle and trip characteristics associated with elevated CO_2_ concentrations in passenger vehicles. They concluded that most one- or two-occupant trips of average duration would not exceed 2500 ppm (ppm) of CO_2,_ a threshold that has consistently been found to impair mental performance. However, for multiple passenger or long-distance trips, the authors suggested that recirculation mode should be periodically interrupted to avoid having CO_2_ concentrations exceed 2500 ppm.

## Discussion

The peer-reviewed literature on individual- and household-level interventions has expanded considerably in recent years. There is compelling evidence from randomized studies that HEPA filter air purifier use over days or weeks can reduce PM_2.5_ concentrations and improve subclinical cardiopulmonary health. There have been fewer studies of facemask use in non-occupational settings, but there is evidence that well-fitting N95 respirators can reduce PM_2.5_ exposure, with several randomized crossover studies also indicating that short-term use improves subclinical cardiovascular health. The effectiveness of other types of masks and face coverings in reducing exposure is highly variable, depends largely on fit, and is unrelated to cost. At present, there is no direct evidence that these masks provide health benefits. Some behaviors—such as traveling on less polluted routes, driving with windows closed, or using enhanced vehicle filtration—may reduce exposure, but there is little evidence that these changes benefit health.

**Table 1:** Summary of publications on individual- and household-level air pollution interventions described in this review

Intervention	Study	Publication year	Location	Study population	Description	Key concentration or exposure results	Key health results
*Air purifiers*							
Portable HEPA	Shao et al. [53], Liu et al. [54]	2017, 2018	Beijing, China	35 older adults	2 weeks each with HEPA and sham filtration in random order	PM_2.5_ reduced from 60 to 24 μg/m^3^; BC reduced from 3.9 to 1.1 m^−1^ × 10^−5^	59% reduction in IL-8. No changes in BP, HRV, or LF.
Portable HEPA	Cui et al. [55]	2018	Shanghai, China	70 young adults	13 h each with HEPA and sham filtration in random order	PM_2.5_ reduced from 33 to 10 μg/m^3^; UFP reduced from 5900 to 2300/cm^3^	7–20% reductions in airway resistance. 27% reduction in VWF. No changes in spirometry.
Portable HEPA	Li et al. [56]	2017	Shanghai, China	55 university students	9 days each with HEPA and sham filtration in random order	PM_2.5_ reduced from 53 to 24 μg/m^3^	Several stress hormones were elevated 1.2- to 1.6-fold during sham filtration compared to HEPA filtration.
MERV 11 filter in window air conditioners	Chuang et al. [57•]	2017	Taipei, Taiwan	422 adults	1 year each with true and sham filtration in random order	PM_2.5_ reduced from 22 to 13 μg/m^3^	Significant reductions in SBP, DBP, and 8-OhdG. Non-significant reductions in CRP and fibrinogen.
Portable HEPA	Barn et al. [58, 59•]	2018	Ulaanbaatar, Mongolia	463 pregnant women	Randomly assigned HEPA or no filter during pregnancy	PM_2.5_ reduced from 25 to 17 μg/m^3^	No effect on birth weight in full study population. Mean term birth weight increased by 85 g.
Portable filtration	Morishita et al. [60]	2018	Detroit, USA	40 older adults	3 days each with sham, low efficiency (LE), and high efficiency (HE) filtration in random order	PM_2.5_ exposure: 15.5 μg/m^3^ sham; 10.9 μg/m^3^ LE; 7.4 μg/m^3^ HE	Any filtration reduced mean SBP and HBP by 3.2 mmHg and 1.5 mmHg, respectively.
Portable HEPA	Zhan et al. [61]	2018	Beijing, China	6 participants (no details provided)	48 h each with HEPA and sham filtration, order not specified	PM_2.5_ exposure increased from 51 to 68 μg/m^3^	**–**
Portable HEPA	Barkjohn et al. [62]	2020	Shanghai, China	43 children	2 weeks each with HEPA and sham filtration in random order	PM_2.5_ exposure reduced from 40 to 25 μg/m^3^	**–**
HEPA & EP in HVAC system	Day et al. [66]	2018	Hunan province, China	89 young adults	Removal of either EP or EP and HEPA for 5 weeks	HEPA removal increased PM_2.5_ by 38 μg/m^3^. EP removal decreased ozone by 2.2 ppb.	No effects of HEPA removal. EP removal led to 16% decrease in P-selectin, 3% reduction in SBP.
IG	Liu et al. [67•]	2020	Beijing, China	56 university students	1 week of true and sham purification in random order	PM_2.5_ reduced from 30 to 8 μg/m^3^; negative ions increased from 53 to 60,591/cm^3^	No intervention effects. PM_2.5_ and negative ions both positively associated with urinary malondialdehyde.
IG	Dong et al. [68]	2019	Beijing, China	44 children	5 weekdays of true and sham purification in classrooms in random order	PM_2.5_ reduced by 44%. Negative ions increased from 12 to 13,000 cm^3^	Intervention associated with a 4.4% increase in FEV_1_ and 15% decrease in FeNO. Benefits on HRV offset by negative effects of ions.
*Facemasks*							
N95	Langrish et al. [73]	2009	Beijing, China	15 adults	2-h walks on a predefined route with and without facemask in random order	**–**	Facemask use associated with decreased SBP and increased HRV.
N95	Langrish et al. [74]	2012	Beijing, China	98 adults with coronary heart disease	2-h walks on a predefined route with and without facemask in random order	**–**	Facemask use associated with lower mean arterial pressure and increased HRV.
N95	Shi et al. [75]	2017	Shanghai, China	24 young adults	48 h with and without facemask in random order	**–**	Facemask use associated with 2.7 mmHg decrease in SBP and increased HRV.
KN90 with pump and noise-canceling headphones	Yang et al. [76]	2018	Beijing, China	40 university students	4-h trips on subway with each of 4 treatments in random order: no intervention, facemask, headphones, and facemask + headphones	**–**	HRV improved with all three interventions.
N95	Guan et al. [77•]	2018	Beijing, China	15 young adults	2-h walks along a busy street with authentic and sham facemasks in random order	**–**	Increases in airway inflammation markers were significantly smaller when authentic facemasks were worn.
Cloth, surgical, N95	Shakya et al. [78]	2017	Laboratory	Mannequin	Evaluation of filtration efficiency of 3 cloth masks and 1 surgical mask	For cloth masks filtration of diesel particles ranged from 15 to 57%. Surgical mask filtration was 79%.	**–**
9 masks	Pacitto et al. [79•]	2019	Laboratory	Mannequin	Evaluation of 9 commercially available facemasks	Mask effectiveness for PM_2.5_ ranged from 14 to 96%. Effectiveness for BC and UFP consistently lower.	
9 masks	Cherrie et al. [26]	2018	Laboratory	10 volunteers	Evaluation of 9 commercially available masks in laboratory, then TIL of best 4 on volunteers in diesel exposure chamber	In volunteer tests TIL of BC ranged from 3 to 68%. 2 masks performed poorly because of inadequate fit.	
17 masks	Mueller et al. [69], Steinle et al. [80]	2018	Laboratory	10 volunteers	Evaluation of PM_2.5_ filtration efficiency for 17 facemasks in laboratory, then TIL of best 4 on volunteers	Filtration efficiencies for N95, surgical masks, and improvised masks < 99%, 89–91%, and < 44%, respectively. The TIL of the N95 mask was 9%; other masks ranged from 22 to 35%.	
*Behavior change*							
Closing home windows	Lin et al. [86]	2009	Taipei, Taiwan	40 university students	Windows open during the first 96-h period and closed during the second 96-h period.	PM_2.5_ 27 μg/m^3^ with windows open; 23 μg/m^3^ with windows closed.	Mean DBP and SBP higher with windows open. Indoor PM_2.5_ associated with BP only when windows were open.
Closing home windows & using AC	Lin et al. [87]	2013	Taipei, Taiwan	300 adults	48 h with windows open, 48 h with windows closed and AC off, 48 h with windows open and AC on. Same order for all participants.	PM_2.5_ decreased 3% when windows were closed and 44% when AC was turned on.	HRV improved with both closing windows and AC. Oxidative stress and systemic inflammation markers decreased only with AC.
Closing windows	Reisen et al. [88]	2019	Victoria, Australia	Residents of 21 homes	1-week indoor and outdoor PM_2.5_ measurements; residents completed a diary on indoor sources and window opening.	During smoke plume events closing windows reduced indoor peak concentrations of outdoor PM_2.5_ by 12% to 76%. Leaving windows closed after the smoke event trapped PM_2.5_ indoors and increased concentrations.	–
Travel mode	Cepeda et al. [91•]	2017	–	–	Review of 39 studies	Car commuters had higher exposure to all pollutants than active commuters in 30 (71%) of 42 comparisons. Active commuters had higher inhaled doses than commuters using motorized vehicles.	Commuters using motorized vehicles lost up to 1 year in life expectancy more than cyclists. Physical activity benefits of active commuting outweigh risk from particle exposure.
Travel mode	De Nazelle et al. [94]	2017	–	–	Review of 10 studies from Europe	Pedestrians consistently had lowest PM_2.5_ exposure. Bus, bicycle, and car exposures were on average 1.3 to 1.5 times higher than pedestrians.	–
Travel mode	Kumar et al. [95]	2018	–	–	Review of 85 studies. Comparison between Asia and other regions.	Avg PM_2.5_ for walkers, cyclists, and drivers: 32, 40, and 40 μg/m^3^, respectively. UFP: 5.3, 3.4, 9.9 × 10^4^/cm^3^, respectively. BC: 10, 6, and 18 μg/m^3^, respectively.	–
Walking route	McCreanor et al. [100]	2007	London, UK	60 adults with asthma	2-h walks in a park and on a busy street in random order.	Median PM_2.5_: 12 vs. 28 μg/m^3^; UFP: 18,000 vs. 64,000/cm^3^; EC: 1.3 vs. 7.5 μg/m^3^.	Walk on busy street reduced FEV_1_ by up to 6.1% and FVC by up to 5.4%. Reductions after walking in park were significantly smaller.
Walking route	Sinharay et al. [101]	2018	London, UK	119 older adults	2-h walks in a park and on a busy street in random order.	Concentrations of BC, PM_2.5_, and UFP significantly higher on busy street.	Walk in park led to increase in FEV_1_ and FVC and a decrease in PWV and augmentation index. Beneficial responses were attenuated after walking on busy street.
Cycling route	Park et al. [102]	2017	Sacramento, USA	32 adults	22-km bicycle rides in a low- and high-traffic area. Order of rides not described.	Median UFP: 16,500 vs. 45,000/cm^3^.	FVC increased during low-traffic ride and decreased during high-traffic ride. FEV_1_ increased during low-traffic ride and did not change significantly during high-traffic ride.
Cycling route	Cole et al. [103]	2018	Vancouver, Canada	38 adults	1-h bicycle rides in a low- and high-traffic area in random order.	Median PM_2.5_: 4.4 vs. 5.5 μg/m^3^; UFP 10,300 vs. 15,700/cm^3^	RHI decreased after cycling the high-traffic route and increased after cycling the low-traffic route. RHI changes not associated with air pollutants.
Cycling route	Lonati et al. [104]	2017	Piacenza, Italy	**–**	Researchers cycled a prescribed route twice daily over 2 weeks in July and September and 1 week in December.	Concentrations of BC and UFP on separated lane and “green path” were 1–2 times and 2–4 times lower, respectively, than on the on-road lane. Traffic proximity not associated with PM_2.5_.	**–**
Cycling route	Hofman et al. [105]	2018	Antwerp, Belgium	**–**	Researchers cycled each of 2 routes 40 times.	BC and UFP 300% and 20% higher, respectively, on road traffic route than on a separated bicycle route.	**–**
Cycling route	Jereb et al. [106]	2018	Celje, Slovenia	**–**	Researchers cycled each of 2 routes simultaneously twice in the morning and twice in the evening.	Mean BC on the main and alternative routes were 6.0 and 5.3 μg/m^3^, respectively.	**–**
Cycling route	Merritt et al. [107]	2019	Stockholm, Sweden	**–**	Researchers cycled on 3 busy streets and 3 parallel quiet streets over 4 weeks.	Cycling on busy streets resulted in BC exposures 1.0–1.4 μg/m^3^ higher than quiet streets.	**–**
Cycling route	Brand et al. [108]	2019	London, UK Rotterdam, The Netherlands, and São Paulo, Brazil	–	Researchers cycled six routes (3 sets of origin-destination pairs) in each city 8–14 times.	Alternative route consistently had lower BC concentrations in London. Results were less consistent in the other 2 cities.	**–**
Cycling route	Qiu et al. [109]	2019	Xi’an, China	**–**	Researchers cycled on two routes a total of 29 times.	Cycling on separated bike lanes decreased exposure to PM and UFP by 32% and 37%, respectively, compared to cycling on roads.	**–**
Cycling & driving route	Good et al. [110]	2016	Fort Collins, USA	45 adults	Participants completed 8 days of commuting by car and bicycle on direct and reduced traffic routes.	For cyclists, alternative route reduced BC and CO by 23% and 10%, respectively. For drivers, alternative route reduced BC and CO by 14% and 15%, respectively. No changes for PM_2.5_ or UFP.	**–**
Vehicle ventilation	Tong et al. [115]	2019	Hangzhou, China	**–**	Researchers studied three vehicles under three road conditions: stationary, local road, and freeway. 21 trips total.	Ventilation was a strong determinant of PM_2.5_ and UFP exposure. Vehicle concentrations were < 10% of outdoor concentration with recirculation, but CO_2_ exceeded 4000 ppm within 20 min.	**–**
Vehicle ventilation	Leavey et al. [116]	2017	St. Louis, USA	**–**	Researchers drove a 6-mile and an 18-mile route. Total of 56 trips across 25 days.	Highest exposures with windows open or the fan on; lowest exposures with windows closed or the AC on. UFP more impacted by window opening than PM_2.5_.	**–**
Vehicle ventilation	Kumar et al. [117]	2020	10 cities	**–**	Researchers drove 3–10 runs at 3 times of day in each city under 3 settings: windows open with fan/recirculation off (windows open); windows closed with fan on (fan on); windows closed with recirculation mode on. 540 runs total.	PM_2.5_ and PM_10_ concentrations: recirculation < fan on < windows open	**–**
Vehicle ventilation	Chuang et al. [118]	2013	Taipei, Taiwan	60 adults	Participants commuted for 2 h with air conditioning off (windows open), in outside air mode, and in recirculation mode. Same treatment order for all participants.	PM_2.5_ with AC off, outside air, and recirculation 36, 13, and 16 μg/m^3^, respectively	HRV indices were higher with AC on and windows closed.
Vehicle ventilation & filtration	Yu et al. [119], Yu et al. [120]	2017 2018	Los Angeles, USA	17 taxi drivers	Each driver was monitored 6 h each with no mitigation (NM), windows closed (WC), and windows closed with high-efficiency cabin air filter (WC + HECA). Same treatment order for all participants.	WC + HECA reduced PM_2.5_ and UFP by 37% and 47%, respectively, compared to NM.	No significant associations with urinary malondialdyde.

*AC* air conditioning; *BC* black carbon; *BP* blood pressure; *CO* carbon monoxide; *CRP* C-reactive protein; *DBP* diastolic blood pressure; *EC* elemental carbon; *EP* electrostatic precipitator; *FeNO* fractional exhaled nitric oxide; *FEV*_*1*_ forced expiratory volume in 1 s; *HEPA* high-efficiency particulate air/arresting; *HRV* heart rate variability; *HVAC* heating, ventilation, and air conditioning; *IG* negative ion generator; *IL-8* interleukin-8; *LF* lung function; *MERV* minimum efficiency reporting value; *PWV* pulse wave velocity; *SBP* systolic blood pressure; *TIL* total inward leakage; *UFP* ultrafine particle count; *VWF* von Willebrand factor; *8-OhdG* 8-hydroxy-2′-deoxyguanosine

Despite their potential benefits, these interventions all have limitations. For example, portable air filters may be prohibitively expensive for some families. In our studies, some participants expressed concern about electricity costs and noise [58•]. In addition, air filters are less effective at higher air exchange rates [58•] or for individuals who spend time in other locations [60–62]. In some studies, participants were asked to stay indoors, keep windows closed, or use air filters continuously, so published results may represent best case scenarios [57•, 61, 62]. The benefits of reduced PM_2.5_ concentrations from electrostatic precipitators and negative ion generators may be offset by the harmful effects of ozone and negative ions, respectively [67•, 68]. Respirators designed for occupational use may be unavailable or prohibitively expensive in some settings [69, 126•], a good facemask fit may be unachievable for children and some adults [127–129], and a poor fitting facemask may give a false sense of security [38]. Higher efficiency masks, such as N95, can be uncomfortable and may make breathing difficult [77•, 80]. This may be particularly problematic for those with compromised cardiorespiratory health, a group that is vulnerable to air pollution and therefore among the most likely to benefit from exposure reductions [41, 42]. Masks marketed for use in community settings are not subject to testing or certification, and mask cost does not predict effectiveness [79•]. Staying indoors and closing windows may be less effective without air conditioning [87], may amplify the impact of indoor emissions [89], and must be weighed against other hazards such as heat and decreased physical activity [90, 91•, 130]. The viability of alternate route selection as an exposure mitigation strategy for active commuters, particularly pedestrians, may be limited if the lower exposure route substantially increases the journey distance [131, 132]. Recirculating cabin air in vehicles can elevate CO_2_ concentrations, particularly on multi-passenger and/or long trips [125].

Our review identified several gaps in the literature. With few exceptions, researchers evaluated IHL intervention use over short durations and measured only subclinical health endpoints. We support calls for studies of long-term use and clinically relevant outcomes [133, 134]. In addition, little is currently known about the long-term developmental impacts from use of these interventions by pregnant women. In our UGAAR birth cohort study, we found that portable air purifier use improved fetal growth [59•], and we are currently investigating whether exposure reductions during pregnancy alter developmental trajectories and provide benefits that extend into childhood [135]. There is anecdotal evidence that N95 and equivalent masks are often unavailable in the most polluted locations, so we recommend health studies of the face coverings that are available and used in those settings. In addition, while a small number of studies evaluated comfort and “wearability” of facemasks among children [128, 129], we are unaware of any studies of the exposure or health benefits of facemask use among children in community settings. The standard advice during pollution episodes is to remain indoors and reduce outdoor activities. This advice is largely based on studies showing that infiltration efficiencies for outdoor PM_2.5_ and other pollutants are < 1. But there is surprisingly little direct evidence that these behavior changes provide any benefits. Thus, there is a need for carefully designed studies to evaluate the effectiveness of this routinely suggested behavior change, particularly for health. We recommend the use of randomized designs when feasible, but researchers will need to carefully consider the inherent ethical challenges [136]. For example, as evidence of air purifier effectiveness continues to accumulate, it may become unethical to withhold them from control participants in randomized studies.

A common criticism of IHL interventions is that they shift the burden of environmental protection from governments to individuals [126•]. Polluting industries may also promote these interventions to avoid or delay government regulations. For example, we question the motives of a Mongolian coal company that has promoted the use of facemasks in Ulaanbaatar. We strongly agree that governments are responsible for air quality management, and IHL interventions can never replace sound environmental and public health policies that address emissions over the long term [126•]. However, when evaluating the merits of these interventions, one should consider that 90% of the world’s population breathes pollution above the WHO guideline concentration [1] and that, historically, policy-driven improvements in air quality took decades [8]. Given this slow grind toward clean air, and the ubiquity of the problem, it is unsurprising that individuals try to protect themselves. In addition, some sources of air pollution—such as forest fires—cannot be directly controlled at the source so risks must be mitigated at the household level [136]. Climate change will increase the relative impact of forest fires on air quality [137], and an increasing number of agencies recommend the use of air purifiers during fire smoke events [138].

A second frequent criticism of these interventions is that they may exacerbate environmental injustice [126•]. However, this criticism assumes that the costs of these interventions are borne by users, which has typically been the case [139]. Air purifiers and facemasks could potentially be distributed by public health or environmental organizations in a way that *reduces* inequities. A recent study in Beijing suggested that air filtration can exacerbate or alleviate exposure inequities, depending on how filtration is used [140•]. PM_2.5_ concentrations in 97 city districts were inversely correlated with mean income and percentage of high school graduates, and the authors argued that governments should provide air purifiers in disadvantaged areas to reduce inequities. Numerous papers have discussed emissions reduction strategies to improve equity [141–143], but the potential for targeted IHL interventions to improve equity has received little attention.

We believe that the research community should rigorously evaluate these interventions so that public health officials can make evidence-based recommendations that enable the public to make informed choices. In addition, as we have described previously, randomized “intervention studies” do more than test interventions; these studies provide large exposure gradients with which to study exposure-health relationships, generate compelling evidence of causality, and provide results that are easily communicated [136].

## Conclusions

There is now substantial evidence that HEPA filter air purifiers reduce indoor PM_2.5_ concentrations and improve subclinical cardiopulmonary health indicators. Several studies have also found subclinical cardiovascular health benefits from well-fitting respirators, while the exposure benefits of other facemasks is highly variable and health benefits have not been tested. Similarly, some behavior changes may reduce exposure but evidence of health benefits remains very limited. Risk mitigation at the household level may be the primary option when emissions cannot be controlled at the source, such as during forest fires, or in communities like Ulaanbaatar where acceptable air quality is seemingly many years away. In most cases, however, individual- and household-level interventions should be supplemental to government policies targeting pollution emissions that benefit entire communities.
